# Development of guidelines for managing unused and expired medications in local communities: An engaged stakeholder waste hierarchy approach

**DOI:** 10.1371/journal.pone.0343225

**Published:** 2026-03-06

**Authors:** Patranit Srijuntrapun, Kusawadee Maluangnon

**Affiliations:** 1 Department of Education, Faculty of Social Sciences and Humanities, Mahidol University, Nakhon Pathom, Thailand; 2 Department of Pharmaceutical Care, Faculty of Pharmacy, Thammasat University, Pathum Thani, Thailand; Mizan-Tepi University, ETHIOPIA

## Abstract

**Objectives:**

The management of unused and expired medications is essential to address environmental pollution, ensure public health safety, and optimize healthcare resources. Proper disposal of medications is especially challenging in areas where there is a lack of organized take-back programs. To bridge this gap, this study aims to develop inclusive guidelines through collaborative stakeholder engagement, while recognizing the absence of universally applicable solutions.

**Methods:**

The study uses a mixed-methods approach, relying on surveys, in-depth interviews, and focus groups. First, 156 households were surveyed to measure the amount and economic value of unused and expired medications in their possession. Subsequently, in-depth interviews were conducted with 45 participants, including healthcare professionals, community leaders, citizens, and policymakers. These in-depth interviews provided rich insights about the roles, local contexts, and challenges regarding the management of unused and expired medications. Lastly, focus group discussions with 16 participants provided the basis for the development of a new set of guidelines aligned with waste hierarchy principles and founded on a holistic view of the medication cycle, from acquisition to disposal.

**Results:**

The findings of the study have revealed that blood glucose lowering agents are the most common type of unused medication kept by households, while anti-hypertensive drugs are the ones that most frequently expire. In addition, the study has shown that medication wastage occurs at multiple stages across the system. Building on these insights, a set of guidelines and community-based management pathways were developed, offering a practical and scalable model in seven steps that can be applied to environments with no formal take-back systems.

**Conclusions:**

The guidelines presented in this study offer a practical and effective framework for policymakers, researchers, and practitioners focused on regions that lack formal disposal mechanisms to guide community-based medication wastage reduction and disposal practices, supporting the sustainable production and consumption of medications.

## Introduction

The appropriate disposal of unused and expired medications has emerged as a pressing concern both in terms of public health and environmental sustainability. The hazards of inappropriate disposal include the pollution of soils and water bodies. Pharmaceutical compounds have the potential to permeate ecosystems, negatively impacting aquatic life and infiltrating the food chain [1]. Addressing these challenges with responsibility is thus of paramount importance.

In 2022, global expenditure on medicines reached approximately $1.48 trillion, a substantial increase from the estimated $887 billion in 2010. Projections indicate that this figure will reach over $1.9 trillion by 2027 [2]. Thailand has also experienced a surge in pharmaceutical consumption, which has grown from Baht 86,544 million ($2.4 billion) in 2008 to Baht 180,585 million ($5.1 billion) in 2018 [3]. Such a high rate of growth, 10–20% above that of developed nations, is attributed to the expansion of medication access in the country, facilitated by universal health coverage. As the population of Thailand has continued to age, chronic illnesses have become more prevalent, further exacerbated by the negative consequences of inappropriate and excessive use of medications.

Data from the World Health Organization indicates that more than 50% of medications are inappropriately consumed in developing countries. This includes improper use of antibiotics, excessive reliance on injectable drugs, concurrent intake of multiple pharmaceuticals, and deviations from prescribed treatments. Such misuse increases the risk of adverse effects on patients and imposes substantial financial burdens on the healthcare system. Pharmaceutical waste can emerge at multiple stages along the medication pathway, including procurement, prescription, dispensal, storage, monitoring, and end-user practices [4]. Importantly, these upstream and midstream processes can substantially influence the volume of unused or expired medications that ultimately require disposal. As most studies tend to concentrate on the behavior of households, these broader system-level drivers remain insufficiently explored. Recognizing such sources of waste is therefore essential for developing a more comprehensive and effective pharmaceutical waste-management model. The World Health Organization has consistently advocated for a judicious consumption of pharmaceuticals and has provided guidelines to promote their rational use among member states.

Despite these global efforts, however, improper disposal of medications remains a critical challenge. Previous initiatives, like the Return of Unwanted Medication (RUM) program implemented by the U. S. Food and Drug Administration [5], a similar RUM project in Australia, or the ENVIRx program in Canada [6], have relied on centralized disposal systems. Many countries, however, have yet to implement programs of this kind. In addition, these initiatives may not suit all regions or communities. Even where countries have adopted them, these programs are often misaligned with regional or community-specific needs or have negative effects on the local environment. While the emphasis of these initiatives is the proper disposal of medications, achieving an effective community management model may involve a spectrum of actions, including preventive measures, reductions in consumption, and more effective methods of disposal. To be successful, these initiatives require critical research that addresses existing gaps related to the management of unused and expired medications.

This study aims to address such gaps by proposing a comprehensive approach that involves the participation of stakeholders from various sectors, including healthcare providers, pharmacies, municipalities, regulators, and the public, in guiding household decisions on appropriate drug disposal. By using the waste hierarchy concept, the study emphasizes the importance of prioritizing waste management strategies aimed at reducing, reusing, and recycling medicines. The aim is to ensure that the disposal of pharmaceutical waste is only a last resort. This integrative methodology represents a novel contribution, as it moves beyond centralized disposal programs to establish system-wide, stakeholder-driven pharmaceutical waste management processes aligned with the waste hierarchy. This ensures that every stage in the life of medications, from generation to final disposal, is coherently addressed within the community. While the study focuses on Thailand, its conclusions may have potential applications in other developing countries.

## Literature review

### Stakeholder participation

According to stakeholder theory, as initially proposed by R. Edward Freeman in 1984 [7], any groups that may experience adverse or favorable consequences from a proposed intervention or who possess the capacity to influence the intervention’s ultimate outcome can be considered as stakeholders [8].

Originally applied to private organizations, the concept of stakeholder has been extended to the public sector [9]. In this context, organizations have been given guidelines for taking into account and balancing the benefits and drawbacks for all stakeholders, including employees, customers, suppliers, local communities, and even the natural environment. This broad perspective recognizes that stakeholders can influence decisions, bear risks, and contribute resources with the aim of creating mutual benefits [10].

Understanding stakeholder relationships contributes to allocate resources efficiently [11]. The interests of stakeholders depend on their knowledge and perception. These characteristics are crucial components of stakeholder theory, which suggests that knowledge of environmental aspects can foster positive ecological behaviors [12]. While some argue that knowledge of sustainability may not always translate into action [13], stakeholder involvement in decision-making remains crucial for long-term sustainable behavior. It enhances transparency, builds trust, and promotes sustainable and responsible practices, fostering diverse thinking and expertise exchange. Moreover, participation facilitates effective engagement and communication, emphasizing the importance of knowledge sharing in achieving goals. Beyond scientific gatherings, the dissemination of knowledge encourages discussion of environmental issues, leading to affective commitment and influencing collective behavior [14].

Applying stakeholder theory to the management of unused and expired medications can provide tangible benefits, particularly when local communities, government agencies, waste collection companies, and environmental organizations are involved in waste management strategies aligned with shared values and goals [15]. This inclusive approach can also ensure that waste management practices are aligned with the environmental needs of the community.

### Waste hierarchy

The waste hierarchy concept, established by the European Union’s Waste Framework Directive (1975/442/EEC) in 1975, prioritizes the adoption of the greenest and most sustainable waste management methods, emphasizing reusing and recycling of products to minimize waste. Since its establishment, the concept has continued to evolve. In 2020, the EU revised it to highlight prevention as the top priority, followed by preparation for reuse. The new hierarchy also includes separate levels of hazardous waste, reflecting the transition to a circular economy that seeks to minimize waste and promote the efficient use of resources [16].

This hierarchy has been applied across various waste categories, energy mixes, and treatment efficiencies [17]. The United States Environmental Protection Agency proposed a Food Recovery Hierarchy [18], while Cole et al. [19] introduced waste hierarchy for the management of electrical and electronic devices, stressing the importance of designing, reusing, recycling, and recovering energy and materials, with disposal remaining the last resort.

The waste hierarchy concept has also been applied to drug waste management in many places. Developed countries like the United States [5], the United Kingdom [20], or Australia [21] promote correct disposal methods through drug recovery programs and government websites. These initiatives often involve designated drop-off points, such as police stations or public hospitals. Rather than aiming at the reduction of drug waste, the primary focus is to ensure the safety of children and other individuals by emphasizing the proper use and secure storage of medicines. Some developing countries have implemented similar programs, even if they are often not mandatory. In Malaysia, for instance, Medication Return Programs (MRPs) encourage citizens to return unused medicines to public hospital pharmacies. Private pharmacies, on the other hand, often refuse to accept them, leading to their improper disposal as household waste [22].

Aligning the management of unused and expired medications with the waste hierarchy can also pose challenges, particularly because the reduction of prescriptions contradicts the hierarchy’s goal to minimize waste. It is difficult to reduce prescriptions considering the crucial role that medicines play in protecting human health. Rational drug management may be necessary to address this challenge. Moreover, while the issue of reuse is critical in the waste hierarchy, giving waste medications to other users can have unintended negative effects on public health. Recalling unwanted drugs is therefore one of the few possible solutions to reduce shortages of medicines [23].

Taken together, the concepts of stakeholder participation and waste hierarchy provide a comprehensive foundation for addressing the challenges of managing unused and expired medications. Although these frameworks have been discussed in prior studies, their practical, community-level implementation remains limited, especially in developing nations. In practice, they have been mostly applied to isolated components rather than to the entire waste-hierarchy system. Based on these perspectives, this study proposes a stakeholder-driven, system-wide strategy aligned with the waste hierarchy as a way to address existing gaps. By moving beyond centralized disposal programs, the context-specific strategies articulated in this study can integrate all stages of pharmaceutical waste management, having both theoretical and practical applications.

## Methods

### Study area and period

The municipality of Ayutthaya in the province of Phra Nakhon Si Ayutthaya is located in central Thailand and has a population of 47,747. This municipality was selected for the study because it is a major tourist destination that has been recognized as a UNESCO World Cultural Heritage Site since 1991. Furthermore, Ayutthaya is one of the provinces with the highest levels of residual waste in Thailand, making it particularly susceptible to environmental contamination from improper disposal of unused and expired medications. The municipal government of Ayutthaya plays a pivotal role in the province’s waste management system, overseeing waste disposal and sanitation. Additionally, community health volunteers (CHVs) from local communities act as change agents, promoting healthy behaviors and disseminating public health information. Their efforts are crucial in fostering proper disposal of unused and expired medicines, significantly contributing to effective management of pharmaceutical waste in the region.

The study was conducted between November 2019 and March 2020. A mixed-methods research approach was employed. Stakeholder theory was used as the conceptual framework to investigate those actors that have the capacity to influence or be influenced by the pursuit of organizational objectives, as defined by Freeman [10]. In addition, the study integrated the principles of waste hierarchy to contextualize the management of unused and expired medications within the environmental sustainability paradigm. The study was developed in three distinct phases: (1) a survey of the quantity and cost of unused and expired medications, (2) a study of the role of stakeholder groups involved in the management of unused and expired medications, and (3) the formulation of appropriate guidelines for managing unused and expired medications at the community level.

### Data collection and characteristics of participants

The data collection process was conducted in three stages:

**Stage 1: A survey of the quantity and cost of unused and expired medications**

*Sample size determination:* From a total of 156 households in the selected community, 112 households were sampled using the Taro Yamane formula [24]. The study achieved a 100% response rate, with all 112 selected households completing the survey.

*Sampling procedure:* To systematically categorize the data, the researchers conducted a survey of the quantity and cost of unused or expired medications held by a sample group comprised of representatives from various households in a particular community of the Ayutthaya municipality. This community was randomly chosen through lottery sampling from a pool of 65 communities. These households were further categorized into three distinct populations: (1) households with chronic patients who are hospitalized or undergoing treatment, (2) households with patients being treated in a hospital for general illnesses, and (3) households with patients having common diseases requiring the purchase of drugs from pharmacies. These three categories are known to differ in their pattern of unused and expired medication management, as shown by previous studies [25,26]. This grouping, therefore, captures heterogeneities in medication use and disposal, reduces clustering bias, and strengthens the representativeness of the sample [27]. The final sample included 59 households with chronic patients (52.7%), 27 households with general-illness patients (24.1%), and 26 households that purchased medicines from pharmacies (23.2%). The categorization in three household types aimed at ensuring an adequate description of the diverse patterns in the use of medications rather than to support inferential group comparisons. Accordingly, no statistical tests (e.g., ANOVA) were conducted during this first stage. The data collection was conducted through a questionnaire, which included the following variables: (1) general demographic information of the sample group, such as gender, age, medical condition, and source of medication; (2) unused medications held in the household; and (3) expired medications held in the household. The questionnaire was developed and thoroughly reviewed by three experts to ensure the validity and suitability of its content.

**Stage 2**: **A study of the role of stakeholder groups involved in the management of unused and expired medications**

*Sample size determination:* A targeted purposive sampling method was employed to identify a total of 45 stakeholders. Sampling was conducted up to saturation by analyzing the collected data to identify the point at which additional data collection was not providing new themes.

*Sampling procedure:* The researchers studied the role of stakeholder groups involved in the management of unused or expired medications by relying on in-depth interviews as the primary tool to categorize information. The sample group comprised stakeholders directly involved in the oversight of unused or expired medications. This diverse group included staff of government hospitals, community health officers, official MCOs, community leaders, pharmacy owners, local administrative officers, as well as representatives from households in the three categories identified above. Each interview group consisted of five individuals. Interviews were conducted using guidelines for semi-structured interviews developed through a literature review on pharmaceutical waste. These guidelines were revised based on the recommendations from experts in qualitative studies to ensure their effectiveness. Open-ended questions were employed to provide interviewees with ample opportunities to fully and freely express their opinions, facilitating a comprehensive understanding of the issues at hand [28]. A purposive sampling approach, coupled with snowball sampling, was employed to ensure diversity across participants in terms of demographic characteristics such as gender, age, and medical condition.

**Stage 3**: **The formulation of appropriate guidelines for managing unused and expired medications at the community level**

*Sample size determination:* The sample size was determined based on stakeholder representation and the objective of achieving consensus. A total of 16 participants were recruited to work on the development of comprehensive guidelines for the effective management of unused and expired medications within the community.

*Sampling procedure:* The researchers created appropriate guidelines aimed at improving the management of unused and expired medications by communities. Group discussions were employed as a strategic tool to gather pertinent information. The sample group, comprised of stakeholders who were actively engaged in the management of waste medicines in the Tha Wasukri community, was randomly selected in alignment with the objectives outlined in the previous stage. These 16 participants were categorized in two separate groups: (1) government hospital staff, community health officials, and MCO staff, as well as community leaders, pharmacy owners, local administrative officers, and public groups (7 individuals in total); and (2) representatives from households that included hospitalized chronic patients, hospitalized patients with general illnesses, or patients with common diseases requiring the purchase of drugs from pharmacies (9 individuals in total). For the final stakeholder meeting, a trained facilitator led a structured two-hour session held at the Tha Wasukri Community Hall. The meeting included guided discussions, the clarification of differing viewpoints, and a step-by-step synthesis of the proposed guidelines. Participants did not receive financial compensation, but they were provided with snacks and drinks. A modified consensus approach was used: key issues were discussed openly, areas of disagreement were documented, and agreements on each component of the guidelines were sought through group deliberation until no major objections remained [29,30].

### Data analysis

The first stage of the project focused on surveying the quantity and cost of unused and expired medications. Quantitative data analysis was conducted to generate descriptive statistics, including frequencies and percentages. The aim was to achieve a detailed understanding of the perspectives of respondents on various aspects linked to the disposal of unused and expired medications. In subsequent stages, content analysis was used to scrutinize primary data derived from group discussions. The data was transcribed, encrypted, and thematically analyzed. Codes were systematically applied and notes were meticulously taken to encapsulate emerging concepts, facilitating the systematic grouping of content in inductive themes. After gaining familiarity with the data, the researchers generated the codes and formulated and named the themes, leading to the preparation of a comprehensive report. The process of data collection, coding, and interpretation was further reinforced through iteration. Two external experts validated the outcome of this process to ensure the robustness and reliability of the resulting data [31].

To minimize biases, credibility was systematically developed in various ways. The researchers took field notes in a reflective journal that aimed to enhance information transfer. Emphasis was placed on completeness and content reliability by establishing sample sizes based on saturation. To ensure the accuracy of meaning, the analytical units used were sentences rather than individual letters or words [32].

### Ethical statement

This research project was undertaken in accordance with existing ethical guidelines and received approval from the Human Research Ethics Committee of Mahidol University (Certificate of Approval No: 2019/133.0406). Before enrolling in the study, potential participants were provided with comprehensive information delineating the objectives and methods of the research. All participants were given written informed consent forms ahead of the data collection process and their explicit consent to participate in the study was obtained. The information collected during the study was treated in strict confidentiality and the researchers used it solely for the purpose of this research.

## Results

### Quantity and cost of unused and expired medications

A total of 112 respondents participated in the survey, including 64 females (57.1%) and 48 males (42.9%). Most respondents were aged between 51 and 60 (26.8%) or between 61 and 70 (26.8%). A total of 59 individuals (52.7%) had household members with chronic illnesses that required hospitalization or ongoing medical treatment. In addition, 27 respondents (24.1%) had household members with general illnesses that required hospitalization or ongoing medical treatment. Finally, 26 households (23.2%) included patients who had general illnesses that required the purchase of medicines from pharmacies. Most participants obtained their medications directly from hospitals (67 individuals, 59.8%). A full breakdown of this demographic data is presented in Table 1.

**Table 1:** Demographic characteristics of participants.

Demographic Characteristics	Number	%
**Gender**		
Male	48	42.9
Female	64	57.1
Total	112	100.0
**Age**		
Up to 40 years	8	7.2
41–50 years	14	12.5
51–60 years	30	26.8
61–70 years	30	26.8
71–80 years	19	16.9
Above 80 years	11	9.8
Total	112	100.0
**Medical condition**		
Chronic patients hospitalized or under treatment	59	52.7
Patients with general illnesses in hospital or under treatment	27	24.1
Common patients purchasing drugs from pharmacies	26	23.2
Total	112	100.0
**Source of medications**		
Hospitals	67	59.8
Community health care centers	12	10.7
Pharmacies	33	29.5
Total	112	100.0

The survey identified some notable practices related to the disposal of unused medications. While some participants (23 individuals, 20.5%) returned unused medications to the hospital, a significant proportion (24 individuals, 21.4%) discarded them. Amongst these, the most common method of disposal was to throw them into household trash bins (15 individuals, 62.5%).

Regarding the management of expired medications, 59 respondents (52.7%) discarded them within the community, while 26 (23.2%) disposed of them outside the community and 17 (15.2%) returned them to the hospital. Among those who discarded expired medications, the most common method (14 individuals, 23.7%) was to throw them into household trash bins. Interestingly, 6 participants (10.2%) discarded expired medications by throwing them into community health care bins, while 2 others (3.4%) used general trash bins or red bins available in the community (See S2 File).

**Fig 1:**
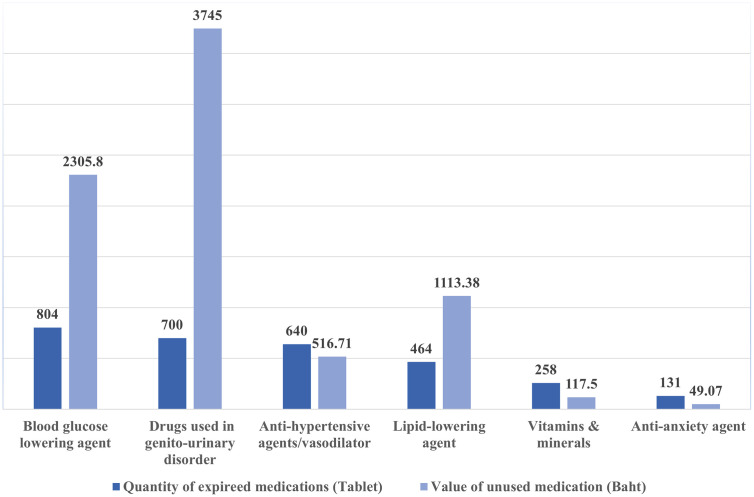
Quantity and cost of unused medications. Note: Drug values are calculated using the average drug price and the normal procurement reference price given by the Ministry of Public Health of Thailand, as of 9 December 2019.

The survey classified unused medications in 31 distinct groups. The most prevalent drugs were blood glucose-lowering agents (804 tablets, valued at Baht 2,305.80 or $63.94), followed by drugs used for genito-urinary disorders (700 tablets, valued at Baht 3,745.00 or $103.85), anti-hypertensive agents/vasodilators (640 tablets, valued at Baht 516.71 or $14.33), lipid-lowering agents (464 tablets, valued at Baht 49.07 or $1.36), vitamins and minerals (258 tablets, valued at Baht 117.50 or $3.26), and anti-anxiety agents (131 tablets, Baht 49.07 or $1.36). As shown in Fig 1, the collective worth of the 31 medication types was Baht 13,767.05 ($381.78).

**Fig 2:**
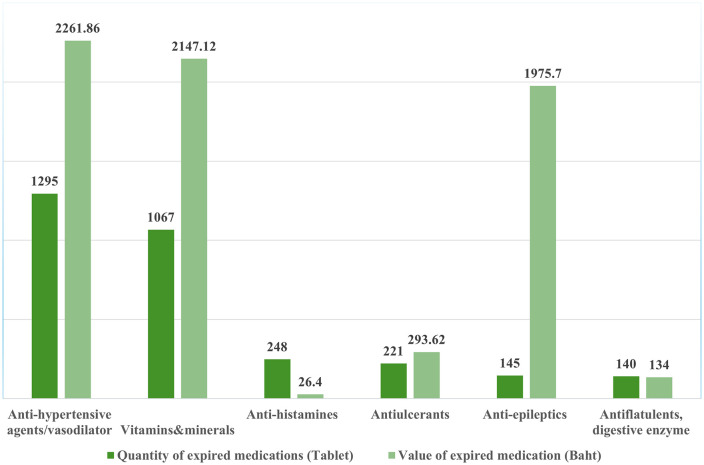
Quantity and cost of expired medications. Note: Drug values are calculated using the average drug price and the normal procurement reference price given by the Ministry of Public Health of Thailand, as of 9 December 2019.

The survey classified expired medications in 29 groups. The most prevalent drugs included anti-hypertensive agents/vasodilators (1295 tablets, valued at Baht 2,261.00 or $62.70), followed by vitamins and minerals (1067 tablets, valued at Baht 2,147.12 or $59.64), anti-histamines (248 tablets, valued at Baht 26.40 or $0.73), anti-ulcerants (221 tablets, valued at Baht 293.62 or $8.14), anti-epileptics (145 tablets, valued at Baht 1,975.70 or $54.79), and anti-flatulents or digestive enzymes (140 tablets, valued at Baht 134.00 or $3.72). As shown in Fig 2, the collective value of the expired drugs in these 29 groups amounted to Baht 9,658.76 ($267.85).

### Roles and responsibilities of stakeholders related to the management of unused and expired medications

This section investigates the responsibilities of the various stakeholder groups and sectors that participate in the management of unused and expired medications. This analysis is undertaken within the framework of the waste hierarchy concept.

***Hospital***: The hospital implements a structured approach to manage its medication system, covering aspects such as supply, dispensal, storage, and routine checks of drug expiration dates. The hospital, however, does not currently facilitate the return of expired or unused medications. Managed as hazardous waste, the safe and proper collection and disposal of expired medications is left to specialized waste disposal companies.

***Community health care center***: The community health care center has a structured management system for medications, akin to the systematic approach employed by the hospital. Regular inspections are conducted to monitor expiration dates and any expired medicines are either rotated out or returned to the hospital to prevent problems. While a system for the return and disposal of unused or expired medications by the public has not yet been implemented, the community health care center plans to use the same red bins that are currently used to collect syringes and other hazardous waste. At the moment, whereas infectious waste is generally transported to the hospital for its disposal, expired medications are being discarded in the general municipal waste and end up deposited in landfills.

***Pharmacies***: Pharmacists employ a systematic approach to manage medications, mostly by coordinating with pharmaceutical companies. This coordination involves the meticulous sorting of medications based on their expiration date, with a mandatory waiting period of at least six months before these medications can be returned to the companies. Despite their systematic approach to drug management, pharmacies do not accept unused or expired medications from the public. Moreover, pharmacies lack a specific system for the disposal of expired medications, relying on methods such as pouring liquids down the drain or tearing red bags (bags for hazardous waste) and tablets to prevent potential resale or reuse before their proper disposal. The hazardous waste in red bags is deposited at the municipal disposal site for collection. In the case of small pharmacies, liquid medications are emptied into toilets or water receptacles, while pills are carefully unpacked and discarded in regular trash bins.

The various responsibilities and tasks of healthcare professionals regarding the management of unused and expired medications are concisely presented in Table 2.

**Table 2:** Roles and responsibilities of the hospital, community health care center, and pharmacies involved in the management of unused and expired medications.

	Hospital	Community health care center	Pharmacies
Medication dispensing system	- Prescribes medication following doctors’ instructions, making minor adjustments as necessary.	- Prescribes medication following doctors’ instructions.	- Dispenses medication in accordance with the customer’s purchase.
Prevention of medication expiry	- Regularly inspects medications. - Organizes soon-to-expire medications at the forefront. - Initiates return to company as needed.	- Regularly inspects medications. - Organizes soon-to-expire medications at the forefront. - Initiates return to hospital as needed.	- Regularly inspects medications. - Organizes soon-to-expire medications at the forefront. - Initiates return to company as needed.
Return system for unused and expired medications	- Return is not required, but unused or expired medications can be deposited at the medicine room.	- Return of unused or expired medications is not required.	- Return of unused or expired medications is not required.
Management of unused medications	- Applicable only to hospitalized individuals. - Donation.	- Current medications are audited to reduce excessive prescription.	- Current medications are audited to reduce excessive purchase of pharmaceuticals.
Disposal of unused and expired medications	- Handed over to hazardous waste disposal company.	- Thrown in the municipal trash.	- Thrown in the municipal trash. - Liquids poured in the drain.

***Community health volunteers*:** The researchers observed that community health volunteers faced challenges when trying to offer suitable guidance for the proper disposal of expired medications due to their limited understanding and lack of prior training on how to handle these medications. As a result, community health volunteers are generally unable to provide the public with appropriate guidance. There is also a noticeable absence of designated facilities for the disposal of these medications.

***Community leaders***: Community leaders seldom engage in efforts to prevent the public from inadequately disposing of unused or expired medications. Given their lack of training, most community leaders do not understand the impacts of improperly handling such materials. This deficiency in knowledge results in a lack of communication on these issues within the community.

***Citizens***: Currently, the community fails to prevent the occurrence of unused or expired medications. Citizens receive minimal information from the government, public agencies, or the private sector concerning the handling of these medications. Consequently, a significant number of people discard expired medications as regular household waste, while unused medications are commonly stored at homes for personal use or for other purposes.

The responsibilities and tasks of community health volunteers, community leaders, and citizens regarding the management of unused and expired medications are outlined in Table 3.

**Table 3:** Roles and responsibilities of community health volunteers, community leaders, and citizens involved in the management of unused and expired medications.

	Community health volunteers	Community leaders	Citizens
Communication efforts to prevent the accumulation of unused or expired medications	- Recommend returning unused medications to medical centers. - Lack of public communication regarding appropriate management of expired medications.	- Recommend returning unused medications to medical centers. - Lack of public communication regarding appropriate management of expired medications.	**–**
Prevention of unused or expired medications	–	–	- Relatively few measures have been implemented.
Return of unused or expired medications	- No action has been taken.	- No action has been taken.	- Relatively few measures have been implemented.
Disposal of unused or expired medications	**–**	**–**	- Thrown in the municipal trash.

Before developing a set of guidelines, a preliminary study was undertaken to evaluate the management of unused and expired medications in the community. The findings of this study are summarized in the SWOT analysis of Table 4.

**Table 4:** SWOT analysis of unused and expired medication management.

**Strengths (S)** 1. The community health care center is ready to collaborate in the supervision of medications, assuming the expenses linked with drug disposal. 2. The community health care center is ready to engage in the promotion and communication of information related to the management of unused and expired medications in the community. 3. Residents in the community are willing to undertake actions to manage unused and expired medications.	**Weaknesses (W)** 1. The public is not properly informed regarding the appropriate management of unused and expired medications. 2. Individuals encounter challenges in identifying the expiration dates of medications for various reasons, such as their lack of comprehension of the English labels and the small font sizes on the packaging. 3. Medical authorities themselves may lack proper understanding of the implications of drug waste on communities and the environment. Furthermore, they often view waste collection as a responsibility delegated to other agencies.
**Opportunities (O)** 1. Communication channels in the community are diverse and efficient. 2. Responsible management of unused and expired medication is part of the objectives outlined in Sustainable Development Goal 12. 3. Properly managing unused and expired medications not only contributes to environmental well-being but also yields economic benefits.	**Threats (T)** 1. There is no specific location designated for the disposal of medications. At present, there is only a hazardous waste bin near or outside the community, which residents cannot easily access. 2. The local waste management system does not encompass pharmaceutical waste, leading to an absence of comprehensive management for this particular type of waste at the source. 3. Society has not given adequate attention and promotion to the understanding of the effects of medicine waste on health and the environment.

### Guidelines for managing unused and expired medications in the community

**Fig 3:**
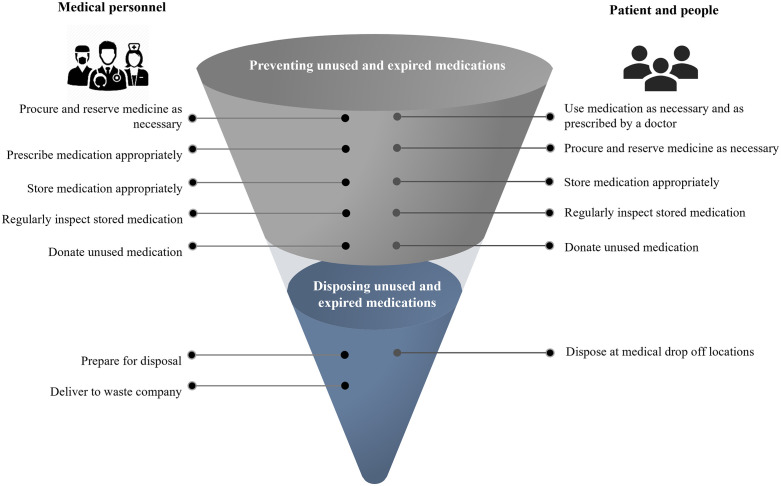
Guidelines for managing unused and expired medications in the community.

The following guidelines for the appropriate management of unused and expired medications are derived from the study of the needs of people in the community and the availability of community resources (strengths, weaknesses, opportunities, and threats), as well as from group discussions. These guidelines are based on the conceptual frameworks of (1) waste hierarchy and (2) rational drug use. Different guidelines are given for each of the stakeholder groups involved in the management of unused and expired medications. As shown in Fig 3, the two main groups considered are (1) healthcare professionals and (2) patients and people in the community.

### Guidelines for healthcare professionals

***Procure and reserve medications as necessary*:** The hospital’s pharmaceutical inventory should be carefully curated, focusing on essential medicines. An annual comprehensive review should be carried out to ensure alignment with quality standards and adequate stock levels. Systematic monitoring of medication quality should be conducted, using collaborative efforts to determine which drugs are essential after consultation with pharmacists, nurses, and physicians.

***Prescribe medications appropriately*:** To prevent medication overuse, it is crucial to avoid overprescribing and preventing the unnecessary extension of treatments. Checking the authenticity of prescribed medications before delivering them is also essential, coupled with assessments and regular follow-ups of patients to comprehensively understand and monitor them. Patients should be advised to bring unused medications when coming for follow-up appointments. They should also be given clear storage guidelines. Emphasis on responsible disposal, especially for high-risk drugs, is vital to mitigate environmental risks.

***Store medications appropriately*:** Medications should have a designated storage space, in adherence to prescribed criteria. Conventional medications should be kept at temperatures below 30°C, while those requiring refrigeration must be stored at 2–8°C. It is crucial to shield stored medications from exposure to red light, and precautions should be taken to avoid proximity to sources of moisture. Additionally, careful management of lighting near the storage area is essential to prevent any negative impacts on drug quality from excessive heat emitted by lamps.

***Regularly inspect stored medications*:** It is imperative to regularly assess the shelf life of medications, both in the sales area and in the storage facility. The implementation of the FEFO (First Expire First Out) system is crucial to ensure that newly received drugs, particularly those with shorter shelf lives, are positioned prominently for immediate use. A robust control system for expired medications, including the use of computers, expired drug notebooks, and color-coded strips, must be established. This system should focus on drugs that will be expiring within the following 6–8 months.

***Donate unused medications*:** A pharmacist or medical professional should thoroughly examine unusued medications for potential reuse, assessing their type, expiration date, and physical quality. Repurposed medications may be distributed to inpatients or donated to healthcare facilities, in alignment with Umphang Hospital’s project for the donation of unused medications. These initiatives enhance access to medical treatment, particularly for individuals without health insurance, promoting equitable healthcare across the country.

***Prepare for disposal*:** A specific area or container should be designated for the disposal of expired or deteriorated medications. These should be marked for return or proper disposal. If returning them is not possible, they should be categorized in three groups: (1) expired or discarded medications should be sorted out, kept in their original containers, and labeled; (2) high-hazard pharmaceuticals like chemotherapy drugs or antibiotics should be kept in a grey bag with appropriate labeling; and (3) specially controlled medications, both modern and traditional, including narcotics, should be stored in a grey bag with specific labeling.

***Deliver to waste company*:** Medical facilities should refrain from directly disposing of unused and expired medications. Instead, they should responsibly deliver them to authorized disposal contractors. This practice is aligned with regulations allowing private entities to operate specialized facilities, as stipulated by the Thai Ministry of Industry.

### Guidelines for patients and people in the community

***Use medications as necessary and as prescribed by a doctor*:** Patients should adhere to prescriptions provided by doctors, especially in the case of antibiotics, disinfectants, and anti-inflammatory drugs. Any medications remaining after finishing a treatment should be taken to the doctor or pharmacist for evaluation. This can potentially reduce costs for the patient. Patients should keep a list of their allergies and inform their doctor and pharmacist during each visit to avoid taking unnecessary medications that may need to be discarded later on.

***Procure and reserve medications as necessary*:** Patients should avoid stockpiling generic medications beyond their needs, as these may expire before being used, resulting in unnecessary waste.

***Store medications appropriately*:** While the majority of medications are typically stored at room temperature, specific drugs like rectal suppositories and certain eye drops should be refrigerated. If the label does not give instructions, patients should refrain from refrigerating medications, as this may result in the accumulation of moisture and the precipitation of water in some drugs.

***Regularly inspect stored medications*:** Conducting routine inspections at least twice a year is crucial to prevent stored medications from reaching their expiration date.

***Donate unused medications*:** Donating unused medications to hospitals or medical centers reduces the overall stock and benefits less privileged patients in remote areas with limited access to drugs. Moreover, this practice aids in curbing the government’s annual expenditure on medical supplies. However, it must be emphasized that sharing or donating medications should be avoided unless a doctor has been consulted.

***Dispose at drop off locations*:** Medications should be appropriately discarded. Patients should refrain from throwing them with the household solid waste, flushing them down the toilet, or discarding them in the sewer. Instead, they should bring them to designated disposal points at the medical center. Discarded medications should be placed separately in appropriate boxes, allowing the medical center to repurpose those that are unused and send the expired ones to hospitals or hazardous waste disposal facilities.

**Fig 4:**
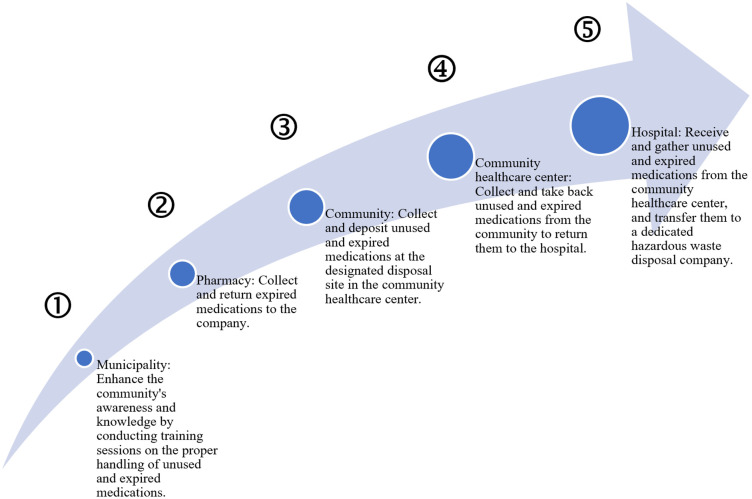
Pathway for managing unused and expired medications in the community.

The pathway for managing unused and expired medications in the community proposed by stakeholders is shown in Fig 4. This visual representation is intended to facilitate comprehension by both patients and members of the community. In addition, the researchers have created an innovative sorting bin for the disposal of unused and expired medications (officially certified under patent no. 18008). After the previous guidelines were made public through booklets and training programs (see Fig 3), there was a notable increase in the number of people dropping unused or expired medications in the new bin. This shows that the guidelines were being used effectively. As a community healthcare provider pointed out, “in a month, people in the community have dropped so many unused and expired medications that the new bin is already full”.

## Discussion

### Quantity and cost of unused and expired medications

The survey presented in this study investigated the management of unused and expired medications in 156 households of the Tha Wa Su Kri community of Phra Nakhon Si Ayutthaya. The study revealed widespread holdings of unused and expired medications, particularly those associated with the treatment of chronic and non-communicable conditions such as hypertension (25.4%) and diabetes (9.5%). These conditions are prevalent among Thai citizens aged 15 years and above, as highlighted in a survey conducted in 2019–2020 [33]. These findings align with previous studies examining pharmaceutical residues in urban waste across various locations, including Bangkok [34], Egypt [35], and Vienna [36], where the most common unused medications were those prescribed for cardiovascular diseases.

Other research studies conducted in India [37] and Japan [38] have also shown that a significant cause of waste is the discontinuation of prescribed medications by patients. Considering that this is an issue that negatively affects patients, a sustainable approach would be to provide accurate information about appropriate medication usage. Moreover, it was found that excessive prescription or instructions to discontinue the use of medications by doctors also tended to increase wastage.

In this study, the cost of unused and expired medications was calculated using the median price stipulated by the Ministry of Public Health of Thailand. This median price represents the government’s expenditure on drugs produced by manufacturers integrated in the public health system. Hence, most of the unused and expired medications surveyed were listed on the National Master List of Medications. In Thailand, patients enrolled in the National Health Insurance System are entitled to receive medications without incurring any costs. Consequently, the disposal of these drugs is economically burdensome for the government. According to the latest data, as of 31 March 2023, there were around 47 million Thai citizens eligible for National Health Insurance or Gold Card under the National Health Security Office (NHSO). In the case of medications that are not included in the national list or that have been acquired from pharmacies for personal use, any economic losses are directly incurred by patients themselves [3].

It is also important to point out that people residing in urban areas have swift and easy access to public health services, a situation that heightens the risk of improper use of medications and contributes to wastage. This factor has a direct effect on the pharmaceutical system, impacting both the supply and the overall value of medications.

### Waste hierarchy and the management of unused and expired medications

Adopting a waste hierarchy approach proves advantageous in the context of managing unused medications, as it offers a systematic way to manage waste from generation to disposal. Such a hierarchy incorporates a preventive stage at the outset, with the aim of minimizing the occurrence of unused and expired medications. Quantitative data from Dutch pharmacies suggests that these preventive measures are effective, having reduced the waste generated from returned medications by more than 30%, with one fifth of these returned drugs deemed suitable for reuse [39].

To ensure the success of measures aimed at mitigating pharmaceutical waste, it is imperative to execute each stage simultaneously, following a careful operational strategy through coordinated efforts among healthcare professionals, patients, and the community [36]. Stakeholder communication, quality assurance, and adherence to legal constraints play a pivotal role in the management of pharmaceutical waste [40]. Some take-back programs depend solely on financial support from the government, while others receive funding from the pharmaceutical industry or from pharmacies operating under the Extended Producer Responsibility (EPR) concept [41]. The launch of PharmaSwap in the Netherlands or the establishment of a website for medicine donations in India [42] are examples of global initiatives in this direction.

The community waste management pathway shown in Fig 4 constitutes an accessible tool that connects community members and facilitates clear communication between them. The waste hierarchy approach for unused and expired medications highlights the importance of cost-effective, user-friendly, and efficient health information delivery, encouraging the development of a comprehensive waste management system from generation to disposal.

The guidelines elaborated for this study were disseminated through printed booklets. To ensure clear and consistent communication of the recommended practices, targeted training sessions were also conducted, both within the community and at the health care center. The fast adoption of the guidelines, as shown by the disposal bins reaching full capacity within one month, provides strong evidence of community acceptance and practical feasibility. In order to scale and sustain such interventions, it is essential to consider their cost, in particular the expenses related to the production of educational materials, the delivery of community training, and the manufacturing of the patented disposal bin.

Although this study has focused on household and community practices, its findings indicate that medication wastage is a system-wide issue that extends across procurement, prescription, dispensal, storage, monitoring, and end-user practices. The guidelines developed here address these various stages. Upstream recommendations, such as selective procurement, FEFO-based inventory management, and avoidance of overprescription, address early contributors to wastage. In the midstream, the guidelines focus on proper storage, regular checks, and organized disposal systems aimed at keeping medications effective and safe. Downstream actions, including safe household storage, regular monitoring, and disposal at designated points, help reduce wastage at the user level.

Qualitatively, the alignment of these recommendations across procurement, prescription, storage, and disposal promotes a system-wide strengthening of medication management. Quantitatively, the increased volume of unused and expired medications collected through the patented disposal bin demonstrates that clear community pathways can enhance disposal compliance. Overall, these guidelines illustrate the potential to reduce pharmaceutical waste throughout the different stages of the medication lifecycle.

The problem of pharmaceutical waste is prevalent in regions without comprehensive take-back programs and effective public awareness initiatives on unused and expired medication management. This is further exacerbated by the lack of budgetary provisions and hazardous waste management systems at the local level. The guidelines for the management of unused and expired medications at the community level proposed in this study can help in crafting a model to effectively handle pharmaceutical waste, addressing local shortcomings.

A limitation of this study is its reliance on self-reported data, as participants tend to report their behaviors based on subjective memories of medication use and disposal. Consequently, future research should employ a combination of methods, including surveys and quantitative data collection from a larger population, which would provide a more comprehensive and accurate overview.

## Conclusions

This study has presented research findings that emphasize the implementation of the waste hierarchy approach in managing pharmaceutical waste. The focus has been on adopting a comprehensive strategy that transcends conventional disposal methods. This approach is perfectly aligned with Sustainable Development Goal 12, which aims at significantly diminishing waste generation through prevention, reduction, recycling, reuse, and proper disposal. The study has advocated for waste reduction throughout the entire product lifecycle, adopting preventive measures during procurement, prescription, and storage, while also promoting the donation of unused pharmaceuticals for reuse. It has also emphasized the need to engage experts and integrate waste hierarchies into pharmaceutical waste management. Moreover, the study has demonstrated that system-wide, multi-stage guidelines spanning procurement, prescription, dispensal, storage, monitoring, and end-user practices can effectively strengthen medication management and reduce wastage, particularly when they are supported by clear community disposal pathways.

Furthermore, the study has practical implications, promoting the implementation of waste hierarchies by local authorities and encouraging patients to return unused medications. This promotes responsible consumption and reduces unnecessary waste. The findings of this study offer valuable insights and actionable steps for policymakers, researchers, and practitioners striving to address medication waste, contributing to the broader global agenda for sustainable production and consumption.

## Supporting information

S1 File Tools used in the study. (PDF)

S2 File Original survey questionnaire for students used in the study. (PDF)
