# Supplementary material for: Development of guidelines for managing unused and expired medications in local communities: An engaged stakeholder waste hierarchy approach
Source: PLoS One. 2026 Mar 6;21(3):e0343225. doi: 10.1371/journal.pone.0343225 (PMC12965569; doi:10.1371/journal.pone.0343225)
Supplement: S1 File — (PDF) [file pone.0343225.s001.pdf]

S1 File. Tools used in the study

This supplementary file includes three research instruments applied at different stages of the study to collect both quantitative and qualitative data.

1. Stage 1: Interview Form on the Quantity of Unused and Expired Medications

- Quantity of Unused Medications

No.	Name of medication:	Quantity
1		
2		
3		
4		

- Reasons for Having Unused Medications
  1. ☐ Received an excessive amount of medication beyond the period until the next scheduled medical appointment
  2. ☐ Did not take the medication continuously as prescribed by the physician
  3. ☐ The physician instructed discontinuation of the medication
  4. ☐ Other (please specify): ___________________________

- How do you manage unused medications in your household?
  1. ☐ Use up the remaining medications before starting newly prescribed medications
  2. ☐ Keep the medications without using them
  3. ☐ Return the medications to a healthcare facility
  4. ☐ Share the medications with others
  5. ☐ Donate the medications through an unused medication donation program
  6. ☐ Dispose of the unused medications

     Please specify the disposal method regularly used:

     ☐ Household trash bin

     ☐ Household toilet

     ☐ Community trash bin

     ☐ Red hazardous waste bin in the community

     ☐ Collection point at a public hospital

     ☐ Community health care centers

     ☐ Other (please specify): ___________________________
  7. ☐ Other (please specify):

- Quantity of Expired Medications

No.	Name of medication:	Quantity
1		
2		
3		
4		

- How do you manage expired medications in your household?

1. ☐ Keep the expired medications without taking any action
2. ☐ Dispose of the expired medications at a hospital or healthcare facility
3. ☐ Dispose of the expired medications
   Please specify the disposal method regularly used:
   ☐ Household trash bin
   ☐ Household toilet
   ☐ Community trash bin
   ☐ Red hazardous waste bin in the community
   ☐ Collection point at a public hospital
   ☐ Subdistrict Health Promoting Hospital
   ☐ Other (please specify): ___________________________
4. ☐ Other (please specify):

2. Stage 2: Semi-structured Questionnaire for In-depth Interviews

1. In your opinion, how do unused and expired medications affect the public or the community?
2. What problems or challenges have you experienced regarding the management of unused and expired medications in the community?
3. What factors or barriers limit your ability to properly manage unused and expired medications?
4. What factors could help promote or enhance the management of unused and expired medications in the community?
5. In what ways can you or your sector provide support, assistance, or resources for the management of unused and expired medications in the community?
6. In your opinion, what would be an appropriate approach to managing unused and expired medications in the Koh Ayutthaya area?

3. Stage 3: Semi-structured Questionnaire for Focus Group Discussions

1. What problems do community members experience regarding the management of unused and expired medications?
2. How do problems related to the management of unused and expired medications affect the community as a whole?
3. Do community members wish to participate in the management of unused and expired medications? If so, how?
4. What factors or barriers prevent community members from adequately managing unused and expired medications?
5. What factors could help promote better management of unused and expired medications in the community?
6. What capacities or resources does each sector have that could support the management of unused and expired medications in the community?
7. What strengths does each sector have that could enhance the management of unused and expired medications in the community?
8. What weaknesses or limitations does each sector have that hinder the management of unused and expired medications in the community?
9. In what ways can each sector provide support or assistance for the management of unused and expired medications in the community?
10. Please analyze and discuss collectively to propose an appropriate action plan and operational framework for managing unused and expired medications in the community.
