# Supplementary material for: Development of guidelines for managing unused and expired medications in local communities: An engaged stakeholder waste hierarchy approach
Source: PLoS One. 2026 Mar 6;21(3):e0343225. doi: 10.1371/journal.pone.0343225 (PMC12965569; doi:10.1371/journal.pone.0343225)
Supplement: S2 File — (PDF) [file pone.0343225.s002.pdf]

S2 File. Figures on the Management of Unused and Expired Medicines

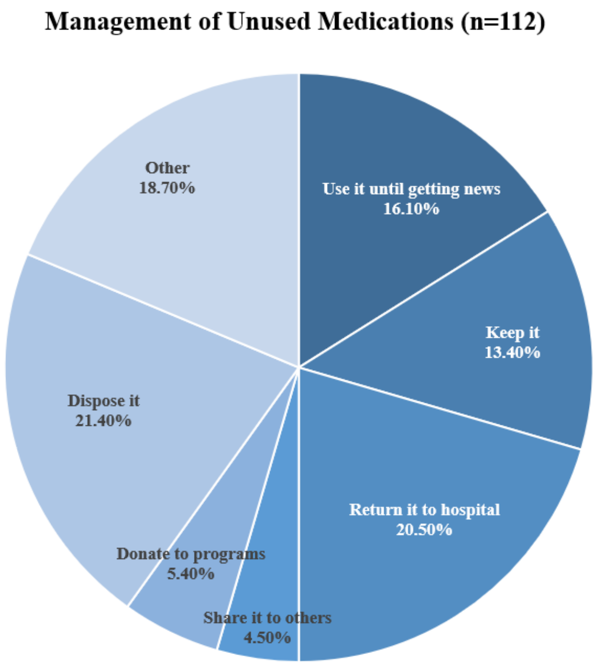

Fig. 1. Management of Unused Medications.

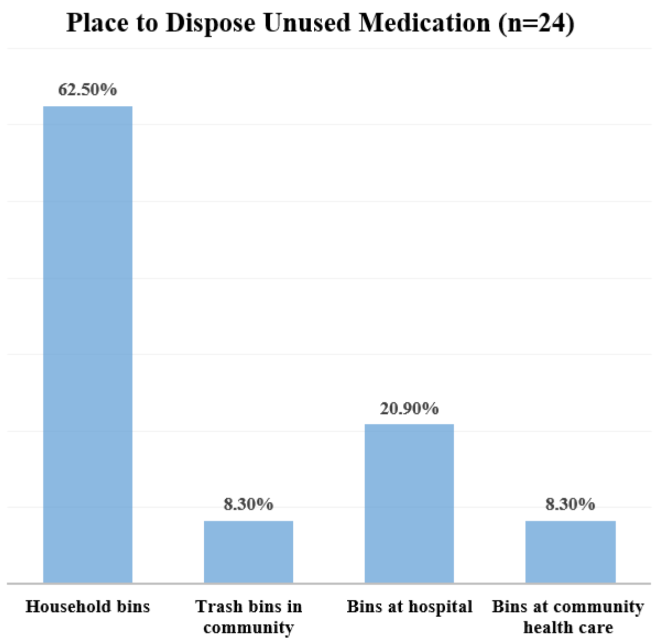

Fig. S2. Places for the Disposal of Unused Medications.

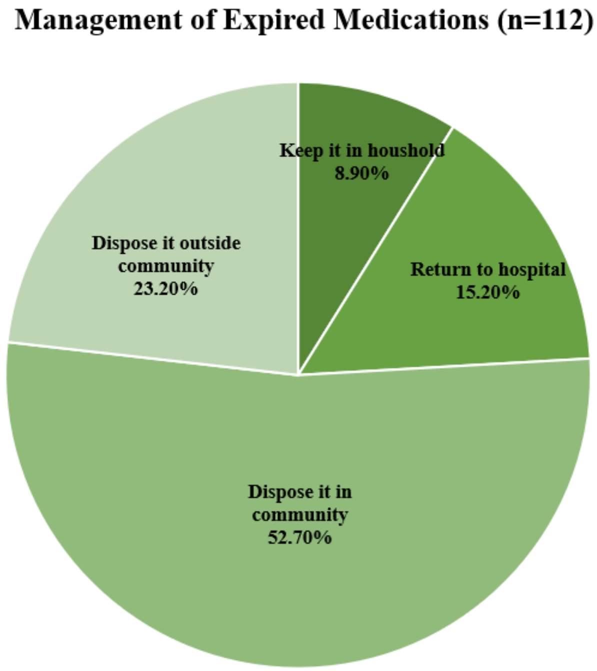

Fig. S3. Management of Expired Medications.

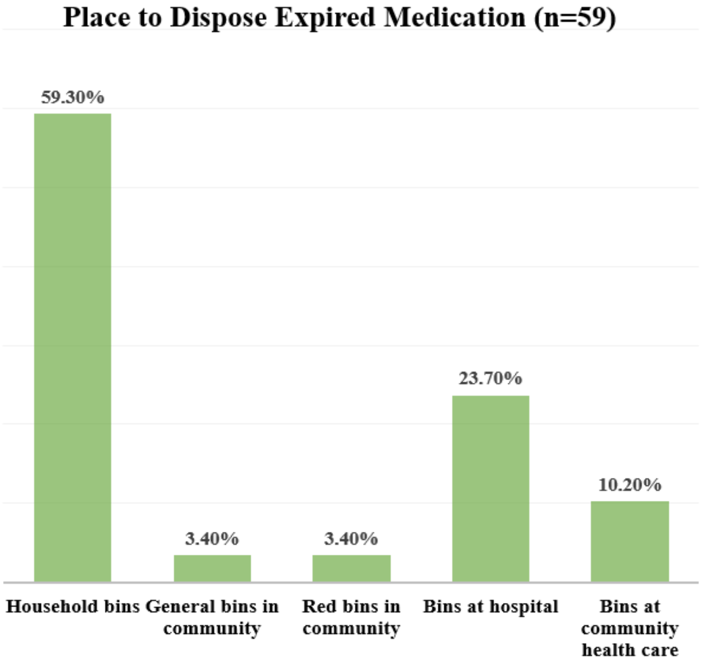

Fig. S4. Places for the Disposal of Expired Medications.
